# ECONOMIC IMPACT OF FRAILTY OR PREFRAILTY IN THE ELDERLY AGED 75 OR OVER IN JAPAN

**DOI:** 10.1093/geroni/igae098.3407

**Published:** 2024-12-31

**Authors:** Hiroto Yoshida, Yuriko Matsuzaki

**Affiliations:** Tohoku Bunka Gakuen University, Sendai, Miyagi, Japan; Japan Health Care College, Sapporo, Hokkaido, Japan

## Abstract

This study examined the impact of frailty or pre-frailty on medical and long-term care expenditures in an older Japanese population. The subjects were those aged 75 years and over who responded to the survey (March 2018) in Bibai, Hokkaido, Japan (n=1,203) and have never received certification of long-term care insurance at the survey. We followed up 758 individuals (63.0%) who used medical services at least once during the follow-up period until the end of August 2019 (17 month-period). We defined frailty as a state in performing 4 items or more and pre-frailty 2 or 3 items of 15 items which were composed of un-intentional weight loss, history of falls, etc. Among 758 subjects, 202 subjects (26.6%) were judged to be frailty group, 210 subjects (27.7%) pre-frailty group, and 346 subjects (45.6%) non-frailty group. We compared period to the new certification of long-term care insurance (LTCI), accumulated medical and long-term care expenditures between the three groups during the follow-up period. Cox proportional hazard models were used to examine the association between baseline frailty and the new certification of LTCI. The relative hazard ratio (HR) was higher in pre-frailty group than non-frailty group (HR=3.93, 95% CI∶1.37-11.22, P=.011). The average cumulative medical and long-term care expenditures per capita during the follow-up period were significantly (P=.025) larger for those in the frailty group (1,190,000 yen). We confirmed the accessibility of pre-frailty to long-term care service use and the strong economic impact of frailty in the elderly aged 75 or over in Japan.

